# Nasal microbiome inhabitants with anti-*Staphylococcus aureus* activity

**DOI:** 10.1128/spectrum.01024-25

**Published:** 2025-09-10

**Authors:** Amy L. Cole, Melissa R. Marzahn, Erika L. Valdespino, Ruchi Patel, Alexander M. Cole

**Affiliations:** 1 Burnett School of Biomedical Sciences, College of Medicine, University of Central Florida 124506, Orlando, Florida, USA; University of Arkansas for Medical Sciences, Little Rock, Arkansas, USA

**Keywords:** *Staphylococcus aureus*, nasal carriage, microbiome, nasal microbiota

## Abstract

*Staphylococcus aureus* (SA) colonizes most mammals but also represents a danger in clinical settings because it evolves resistance against antibiotics, and SA infections represent a leading cause of death worldwide. SA nasal carriage provides the bacterial reservoir for opportunistic infection because clinical strains often match the patient’s own nasally carried strain. The global SA carriage rate is typically reported as 25%–30% after sampling subjects once or twice and defining carrier status using culture-based methods. Here, we collected nasal swabs from 31 healthy subjects twice weekly for an average of 5 months and determined that 23 of 31 subjects (74.9%) presented SA colonies at least once during longitudinal sampling. We explored the nasal microbiota of individuals who rarely or never tested SA-positive and evaluated 25 nasal isolates for anti-SA properties using culture conditions modeling the nasal mucosa. We determined that members of the *Gammaproteobacteria* class, including *Klebsiella* spp.*, Serratia marcescens*, and *Enterobacter hormaechei,* inhibited SA survival. Firmicutes *Dolosigranulum pigrum* and *Streptococcus mitis/oralis* also inhibited SA recovery from nasal tissues. *Klebsiella* spp. (*aerogenes, variicola, pneumoniae*) activity was contact-independent and not mediated by sequestration of iron or other vital nutrients. *K. aerogenes* was the most potent SA inhibitor (>99% reduction), and its action was mediated by secreted cationic peptide(s) or protein(s). Collectively, this project determined that most people may be nasally colonized with SA throughout life. It also represents an important step toward explaining why some hosts don’t exhibit detectable nasal SA and highlights a potential source of naturally occurring and novel antibiotics targeting SA.

**IMPORTANCE:**

*Staphylococcus aureus* (SA) is a common member of the human microbiota, colonizing people transiently throughout life, and SA nasal carriage is an important reservoir for the spread of this bacterium. The global economic toll of SA infections is enormous, and antibiotic-resistant strains continue to emerge. Therefore, new approaches to decolonize carriers of SA are urgently needed. To better understand the dynamics of nasal SA carriage versus non-carriage, we monitored subjects longitudinally and isolated and identified species that associate with SA culture-negative noses. While several members of the *Gammaproteobacteria* class demonstrated anti-SA activity, the nasal *Klebsiella* spp. potently inhibited (>99%) SA survival in a contact-independent manner. For multiple nasal isolates of *K. aerogenes* from our donor cohort, this inhibition was attributed to a small, cationic, proteinaceous molecule(s). The secreted products of nasal *Gammaproteobacteria* (e.g., *Klebsiella*/*Serratia* spp.) warrant further investigation as potential sources of new SA decolonization agents.

## INTRODUCTION

*Staphylococcus aureus* (SA) is both a well-characterized pathogen and a member of the human microbiota that is ubiquitous enough that approximately 70% of infants are colonized between birth and 8 weeks, and nearly all people possess anti-SA immunoglobulins (1–6). The dynamics of SA carriage are complex, as frequency (culture positivity) drops to 20%–30% by 1 year before rising to 50% during adolescence and settling toward an apparent rate of 20%–30% in adulthood (6). SA represents a global threat because it is one of the most isolated pathogens from infected wounds, surgical sites, and cases of severe pneumonia and sepsis (6, 7). SA infections complicate treatment protocols and are associated with increased hospitalization rates, duration, and overall medical expenses; they are major contributors to antimicrobial resistance (6–8). The fatality rate associated with SA bacteremia is approximately 25% (9), even in developed healthcare systems, and SA is now the most common causative microorganism of infective endocarditis (10). Thus, continued efforts toward better SA decolonization strategies are needed.

SA colonizes mucosal surfaces and skin, and SA nasal carriage represents an important reservoir for the spread of the bacteria. For most individuals, the infecting strain is genetically indistinguishable from one’s own nasally carried strain (1, 11, 12); and it is now widely accepted that SA carriage elevates the burden of SA infections because SA nasal carriers are more likely to seek clinical care and antibiotic therapy, and to experience post-surgical infections (11, 13–15). The overall nasal carriage rate corresponds with both host and environmental factors. Atopic dermatitis, HIV, diabetes, and dialysis patients exhibit higher rates than healthy subjects, as do newborns, non-diabetics with BMI >30, industrial farm workers, and people living in close confinement (16–19). Collectively, a 25%–30% SA nasal carriage rate is presumed for all humans at any given moment, based on dozens of studies that sampled subjects once or twice over a short period (12, 20–23). For healthy (asymptomatic) individuals, about 20% are considered persistently colonized (e.g., test positive for SA at most or all longitudinal sampling sessions) while the remaining population appears to acquire and clear SA repeatedly (“intermittent” carriers) or usually presents no SA colonies (non-carriers) when tested and classified via culture methods (21, 24, 25). It is not completely understood whether non-carriers truly exist or whether their colonizing SA is undetectable by established protocols. Numerous host factors are linked with nasal antimicrobial defense; however, elevated host antimicrobial peptide levels associate with SA nasal carriage rather than non-carriage (2, 3, 26–28), and anti-SA antibody profiles are not modulators of colonization status or duration (29). Most SA strains appear able to colonize the nasal mucosa (30–32), and a study of the composition of nasal fluid revealed no apparent nutritional differences between fluids collected from SA carriers versus non-carrier noses (33). This aggregate evidence that host and environmental attributes, such as chronic disease states, dictate the onset and duration of SA nasal carriage, while an appreciable percentage of people don’t carry nasal SA at detectable levels, has finally led the research community to explore the role of the nasal microbiota in elaborating a defense against SA.

Advances in microbial DNA profiling of swab samples have begun to associate SA nasal carriage with bacterial community state types (CSTs), although species-level microbial profiling was rare until recently (34–36). In a study of monozygotic and dizygotic twin pairs, it was determined that host genetics did not shape the nasal microbiota (only 26% of monozygotic twins had the same CST) but did influence nasal bacteria density, and the authors concluded that culture-based methods fail to identify a substantial proportion of SA carriers (34). Likewise, prioritizing live bacteria in sample preparation and metagenomic sequencing workflow revealed that SA DNA is detected in >80% of noses (37). In our previous study of established non-carriers and persistent SA carriers, nasal swabs were analyzed utilizing an approach that combined host (human) DNA depletion with shotgun metagenomic sequencing that enabled species- and strain-level sequence resolution (38). SA DNA was detected in all noses, and some culture-negative nostrils contained a higher relative abundance of SA DNA than some persistent SANC nostrils. Members of the *Gammaproteobacteria* class, including *Klebsiella aerogenes* (formerly known as *Enterobacter aerogenes*), were isolated from noses that contained SA DNA but were deemed culture-negative for SA and were determined to exhibit anti-SA activity in nasal tissues and secretions (38). As a result of a heedless, extended power outage in our laboratory building in 2021, our sample collection was lost to spoilage, and we spent considerable effort enrolling and sampling new subjects, monitoring SANC status, and rebuilding a nasal sample library. Here, we demonstrate with an entirely new cohort that SA culture-negative noses harbor numerous bacterial species with functional anti-SA capacity. Nasal *Klebsiella* spp. (*aerogenes, variicola, pneumoniae*) and *Serratia marcescens,* along with select Firmicutes, including *Dolosigranulum pigrum* and *Streptococcus mitis/oralis*, were found to inhibit SA recovery in support of the concept that nasal CST-2, 6, and 7 predict non-carriage (34). Notably, *K. aerogenes* was the most potent inhibitor of SA survival and was recovered from 60% of culture-defined SA-negative subjects. Secreted products from *K. aerogenes* warrant further investigation and represent a naturally available antimicrobial agent with potential for use as an SA decolonization therapy.

## MATERIALS AND METHODS

### Chemicals, reagents, and preparation of simulated nasal medium

Bacterial broths were purchased from Fisher Scientific, Inc.: Tryptic Soy Broth (TSB, cat# DF0370-17-3), Brain Heart Infusion broth (BHI, cat# DF0418-17-7), Brucella broth (cat# B11088), and Todd Hewitt broth (cat# DF0492-17-6). Hank’s balanced salt solution (HBSS, Fisher cat# 14-025-076) supplemented with 0.1% (vol/vol) bovine serum albumin (BSA, Millipore-Sigma, St. Louis, MO, cat# A7906) was used for diluting, washing, and rapid-freezing bacteria preparations in liquid nitrogen. Tissue culture-grade Dulbecco’s phosphate-buffered saline (PBS) was from Fisher (cat# MT21031CV). Preparation of synthetic nasal medium (SNM) was as described for SNM3 by Krismer et al. (33) based on the metabolomics data from nasal secretions. SNM leads to gene expression patterns similar to those observed during *in vivo* SA colonization (33). All components were purchased from Fisher Scientific or Millipore-Sigma and prepared as stock solutions as described in detail previously (38). For all presented experiments, we utilized SNM3 supplemented with 0.1% (vol/vol) human serum (GeminiBio), termed SNM-HS, to mimic the protein content of nasal secretions (39, 40) and support the growth of diverse nasal bacteria *ex vivo* (38).

### Study population and sample collection

A protocol for the recruitment of healthy participants and collection of nasal swabs was approved by the Institutional Review Board of the University of Central Florida. Participants sampled each nostril by rotating a sterile polyester-tipped swab around the anterior vestibule 10 times. Swab tips were vortexed in 2 mL SNM-HS to liberate the microbes. From each 2 mL swab sample, 0.1 mL was spread onto each of the following agars: BD CHROMagar *S. aureus* (Fisher Scientific cat# 14-432-41) for identification of SA, non-selective TSAII/5% sheep blood agar and Columbia/5% sheep blood agar (Fisher Scientific cat# B21261X and R01522), Columbia CNA agar (Fisher Scientific cat# R01322) for assistance with selecting Gram (+) species, MacConkey II (Fisher Scientific cat# B21270X) for selection of Gram-negative species, and Chocolate II (Fisher Scientific cat# B21267X) for selection of *Streptococcus* species. The remaining volume was used for creating an “early” glycerol stock (0.5 mL swab sample + 0.5 mL 30% glycerol in TSB) and for culturing overnight (37°C/5% CO_2_) in 14 mL round-bottom snap cap tubes. Overnight cultures were again saved as glycerol stocks and, when CFUs were low upon initial plating, plated in 10-fold serial dilutions to the agar listed above. Nasal swabs collected solely for microbial community DNA profiling were processed using the DNA/RNA Shield swab collection kit (Zymo Research, Irvine, CA, cat# R1107).

### Nasal bacteria isolate details and stock preparation

Isolated colonies were cultured in 4 mL bacterial broth (either or all of TSB, BHI, Brucella, or Todd Hewitt) depending on the observed growth of similar isolates throughout the project overnight at 37°C/250 rpm. The next day, 1 mL was supplemented with glycerol to 15% and stored at −80°C, and the rest was centrifuged to collect bacteria prior to DNA extraction (Zymo Research Quick-DNA Fungal/Bacterial Miniprep kit, cat# D6005). Purified DNAs were submitted to Azenta Life Sciences (formerly Genewiz, Inc., South Plainfield, NJ) for microbial identification using full-length 16S rRNA PCR and Sanger sequencing with “best hit” blastn results returned for each DNA. Non-SA nasal isolates identified and stored in our laboratory were *Staphylococcus epidermidis* (eight donor noses), *Staphylococcus haemolyticus* (one donor nose)*, Staphylococcus lugdunensis* (two donor noses)*, Staphylococcus pasteuri* (five donor noses)*, Staphylococcus warneri* (two donor noses), *K. aerogenes* (six donor noses), *K. variicola* (one donor nose), *K. pneumoniae* (one donor nose), *S. marcescens* (two donor noses), *E. hormaechei* (one donor nose), *Escherichia coli* (two donor noses), *Escherichia fergusonii* (two donor noses), *Raoultella ornithinolytica* (one donor nose), *Pantoea rodasii* (one donor nose), *Streptococcus mitis/oralis* (seven donor noses), *Bacillus tropicus* (two donor noses), *Bacillus paramycoides* (one donor nose)*, Bacillus koreensis* (one donor nose), *D. pigrum* (two donor noses), and *Corynebacterium accolens* (four donor noses). The following nasal isolates were purchased from American Type Culture Collection (ATCC, Manassas, VA): *K. aerogenes*-819-56 (cat# 13048), *Cutibacterium acnes* (cat# 6919), and *D. pigrum* (cat# 51524). Vials were cultured according to ATCC instructions, and bacteria from this first overnight culture, as well as several isolated colonies, were stored as glycerol stocks at −80°C. Nasal SAs used in this study were collected from healthy, colonized subjects by our laboratory and genotyped using multilocus sequence typing and *spa* typing as described previously (24, 31, 41). Specifically, the strains were D547 (ST5, t688), D512 (ST30, t012), and D713 (ST5, t548). USA300 (ST8, t008, multi-drug resistant) was acquired from NARSA (now https://www.beiresources.org/). These strains represented a cross-section of the most common sequence types described in our large longitudinal study of over 100 nasal SA carriers (24). Additional nasal SA isolated in this study was collected from six donors. Because liquid culture (37°C/250 rpm) in rich broth (e.g., TSB, BHI, Brucella) is not physiologically relevant to the nutrient limitation experienced by nasal microbiota, isolates (SA and putative competitors) were propagated for use in experiments via culture (24 h/37°C/5% CO_2_) in a 100 mm dish of confluent primary nasal epithelial cells containing 10 mL of SNM-HS. Nasal cell-free, washed bacteria were flash frozen in liquid nitrogen and stored at −80°C in aliquots (0.3–1 × 10^6^ CFU/µL in HBSS/0.1% BSA) for use in experiments.

### Next-generation sequencing of nasal swabs

Forty-eight nasal swabs were submitted for “full shotgun metagenomic service” by Zymo Research Corporation (Irvine, CA), according to company instructions. This service comprised DNA purification (ZymoBIOMICS-96 MagBead DNA kit), library preparation (Nextera DNA Flex Library Prep Kit [Illumina, San Diego, CA]), pooling and post-library QC, Illumina sequencing (NovaSeq [Illumina]), and bioinformatics analysis (42–46). Four of the swabs (two each from two different donors) were eliminated from analysis because the donor SA carriage status was not understood at the time of analysis. The presented data, therefore, summarize microbial reads for 44 swab samples. Total raw reads averaged 16.8 M per sample, with 93% retained/7% dropped. Host reads averaged 6.8 M (92.9%), with 411,608 microbial reads (5.62%) and 108,790 reads unclassified. Zymo did not recommend or perform host DNA depletion, but it is our view that nasal swabs should be depleted of host DNA prior to sequencing based on deeper microbial profiling achieved in our previous study (38).

### Air-liquid interface primary nasal epithelial cell culture

Human primary nasal epithelial cells (NEC, PromoCell cat# C-12620 from Millipore-Sigma) were maintained and expanded on collagen-coated tissue culture plates using Airway Epithelial Cell Growth Medium (base+supplement mix) (Promocell cat# C-21060, from Millipore-Sigma). A 30× collagen solution was purchased from Advanced BioMatrix (San Diego, CA) and diluted to 1× with sterile, tissue-culture grade water prior to each use. Each 100 mm dish was coated with 5 mL of 1× collagen for at least 2 h (37°C/5% CO_2_); then the liquid was removed, and plates were rinsed with 5 mL PBS before cells were seeded. Early passage NECs were maintained in the presence of 1× Primocin (broad-spectrum antibiotic/anti-mycoplasma solution purchased as a 500× mix from Fisher Scientific, cat# NC9141851), and aliquots of cells were frozen and stored in liquid nitrogen for use at passages 3–7. The freeze medium (Millipore-Sigma cat# C29912) and all associated buffers and reagents used for cell maintenance were part of Promocell’s serum-free solutions for primary cells. To prepare for the co-culture of NEC with bacteria at the air-liquid interface (ALI), cells from a confluent 100 mm dish were seeded at 0.5 mL per well in a 12-well PET Transwell dish (Corning# 3460, purchased through Fisher). These 0.4 um pore Transwells were pre-coated with collagen as described above. The day after cell seeding, an ALI medium was prepared by supplementing antibiotic-free maintenance medium with 0.4 mM calcium chloride, and this medium was used for daily medium changes (1 mL per underlay, 0.3 mL per overlay until day 3 or 4, when medium was removed from the apical cell surface). Treatments were performed within 4–7 days following ALI exposure, when transepithelial electrical resistance (TEER) exceeded 300 Ω∙cm^2^ and tissues sealed the basal medium away from the apical compartment of the Transwell, as evident by visual inspection. TEER was measured using the EVOM^2^ voltohmmeter and EndOhm-12 chamber for 12 mm culture cups (World Precision Instruments, Sarasota, FL).

### Co-culture of SA and competitors on NEC Transwells

One day prior to SA competition assays, Transwell underlay medium was removed from each well and replenished with fresh ALI medium, and apical surfaces were confirmed to be moist but devoid of underlay medium that can seep through disrupted epithelia. Leaky Transwells were not used for experiments because excessive seepage of underlay medium into the apical chamber promotes bacterial overgrowth. For screening of competitor species, including *S. epidermidis*, for activity against SA, apical surfaces were pre-treated with 30,000 CFU (~MOI = 1) of a commensal species mix comprised of 15,000 CFU each of *C. acnes* and *C. accolens*, followed by addition of SA and/or competitor species 24 h later. For testing *K. aerogenes* activity with and without iron supplementation, the commensal mix contained 15,000 CFU of *C. acnes*, 5,000 CFU of *C. accolens*, and 10,000 CFU of *S. epidermidis* to mimic the ratio of these commensals observed in our non-SA carrier donor cohort. For all experiments, each bacterial stock (preparation described above) was thawed rapidly by swirling in a 37°C water bath, then diluted with SNM3-HS such that each Transwell was treated with 80 µL commensals, followed by 5,000 CFU of SA or 5,000 CFU SA + 5,000 CFU competitor strain in a total of 100 µL. Serial dilutions of the prepared bacteria were agar plated to record the actual CFU/well at time 0. After 24 h (37°C/5% CO_2_), apical fluids (including disrupted/floating cells) were transferred to microtubes, and the remaining cells were vigorously washed and transferred to the same collection tube using ice-cold HBSS. These apical fluid-rinse mixtures were centrifuged at 300 × *g* (4°C, 5 min) to separate epithelial cellular debris from bacteria. Bacteria were then collected by centrifugation at 10,000 × *g* (4°C, 5 min), suspended in HBSS, and dilution-plated to various agar formulations: CHROMagar SA, TSAII/5% sheep blood, MacConkey II, and Chocolate II (for wells containing *S. mitis/oralis*). Underlays (containing epithelial cell medium and any bacteria that penetrated the tissue) were collected and stored on ice, then agar-plated for bacterial enumeration after the apical fluids were plated.

### Preparation and processing of conditioned medium for evaluation of anti-SA effectors

To prepare conditioned medium (CM) from bacterial cultures, 100 mm tissue culture dishes were inoculated with 10 mL/dish of SNM-HS containing ~5 × 10^6^ CFU of the bacteria of interest (e.g., *K. aerogenes*) and incubated at 37°C/5% CO_2_. After 24 h, each dish was scraped with a cell lifter, and the combined mixture of bacteria and supernatant was transferred to conical tubes. After centrifugation at 10,000 × *g* (4°C, 5 min) to collect bacteria, clarified supernatant was passed through a 0.2 um PES syringe filter (Corning #431229, purchased through Fisher) to remove residual bacteria, and the resulting CM was tested for bacterial growth by direct plating to TSAII/0.5% blood agar plates, and stored in aliquots at −80°C until use. Anti-SA activity of individual CM preparations was evaluated by adding 10 µL (10,000 CFU) of freshly thawed and diluted SA to 490 µL of freshly thawed bacterial CM or control CM in a 24 W tissue culture dish followed by incubation at 37°C/5% CO_2_ for 24 h. Bacteria were enumerated by plating serial dilutions on agar plates, and fold growth was calculated (T_24h_/T_0h(input)_). Buffer-exchanged CMs were manipulated by running clarified CMs through Amicon Ultra 3kD centrifugal filters (Millipore-Sigma cat# UFC500324), according to the manufacturer’s instructions. This was followed by three washes with water and adjustment back to the original volume with fresh SNM.

### Proteomics analyses of conditioned media

Conditioned medium collected from *K. aerogenes* was sent to the University of Florida Interdisciplinary Center for Biotechnology Research, Proteomics and Mass Spectrometry Core Facility (RRID:SCR_019151), and Mascot result files and Scaffold result reports were returned. Research resource identifiers (RRIDs) are unique identifiers for referencing a research resource in support of the Resource Identification Initiative (https://www.rrids.org). Trypsin/Lys-C in solution digestion was performed, followed by liquid chromatography tandem mass spectrometry (LC-MS/MS) with the following instrument method: An Orbitrap Fusion Tribrid Mass Spectrometer system (Thermo Fisher Scientific, San Jose, CA) was interfaced with an ultra-performance Easy-nLC 1200 system (Thermo Fisher Scientific, Bremen, Germany). Each desalted sample was loaded onto an Acclaim PepMap 100 pre-column (20 mm × 75 µm; 3 µm-C18) and eluted using a PepMap RSLC analytical column (250 mm × 75 µm; 2 µm-C18). The flow rate was set at 250 nL/min with solvent A (0.1% formic acid, 99.9% water [vol/vol]) and solvent B (0.1% formic acid, 80% acetonitrile, 19.9% water [vol/vol]) as the mobile phases followed by a linear gradient: 2% of solvent B in 5 min, 2%–35% of solvent B in 70 min, ramping up to 80% solvent B in 5 min to 98% solvent B in 1 min, and isocratic at 98% in 14 min. The mass spectrometer acquired the data under the collision ion dissociation (CID) mode in each MS and MS/MS cycle, scanning from 350 to 1,800 m/z. The maximum ion injection times for the survey scan and the MS/MS scans were 35 ms. MS1 spectra were recorded at a resolution of 120,000 full width at half maximum from 350 to 1,800 m/z with quadrupole isolation followed by one MS/MS scan of the most intense precursor ions in the linear ion trap. The automated gain control (AGC) target was set to 2 × 105, with a max injection time of 50 ms. The quadrupole was used for precursor isolation with an isolation window of 1.3 m/z. Only precursors with charge states 2–6 with an intensity higher than 1 × 104 were selected for fragmentation. The monoisotopic precursor selection filter was activated. The option to inject ions for all available parallelizable time was selected. Targeted MS2 spectra were acquired and were performed in the ion trap with CID fragmentation (Rapid; NCE 35%; maximum injection time 35 ms; AGC 1  × 104). The normalized collision energy (NCE) was set to 35% for each fragmentation method, and one microscan was acquired for each spectrum.

### Statistical analysis

Data were analyzed using GraphPad Prism 10 (v10.5.0 [673]) software (GraphPad Software, La Jolla, CA). In most cases, SA counts (CFU/treatment well) were expressed as percent recovery: SA CFU yield for SA alone (no competitor) was set at 100% and compared to SA yield when admixed with test bacteria or CM generated from competitor species. Groups were compared using ordinary one-way analysis of variance with Dunnett’s multiple comparisons test or unpaired *t*-tests.

## RESULTS AND DISCUSSION

### Longitudinal sampling reveals SA nasal carriage rate exceeding 60% in healthy volunteers

**Fig 1:**
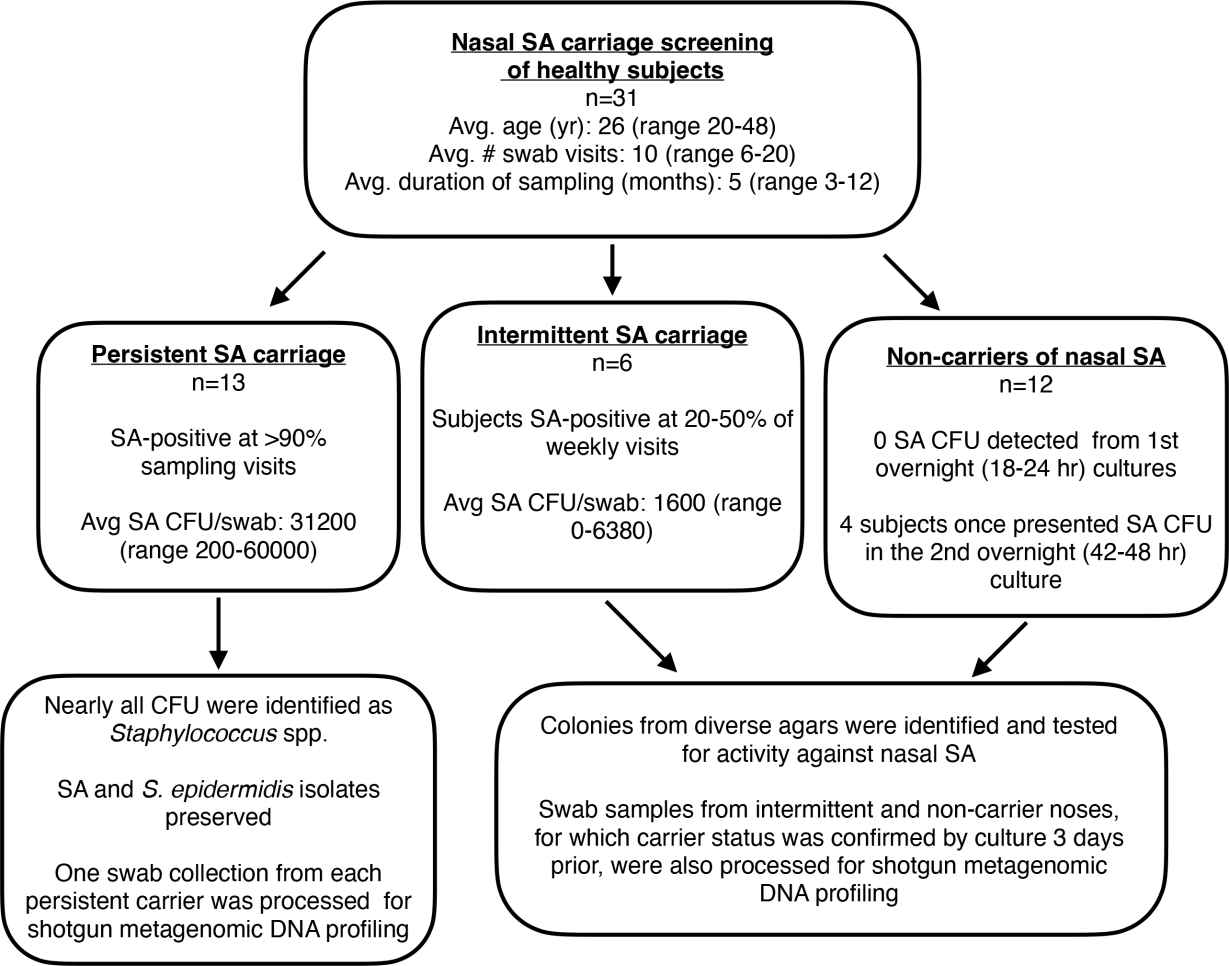
Study population and experimental overview. Participants self-collected nasal swabs and were monitored for SA nasal carriage and classified as persistent, intermittent, or non-carriers based upon detection of SA by culture. Bacterial CFU from intermittent and non-carriers were isolated, identified, and tested for activity against nasal SA. Nasal swab samples from all subjects were also analyzed at the DNA level using a shotgun metagenomic bacterial profiling approach.

**Fig 2:**
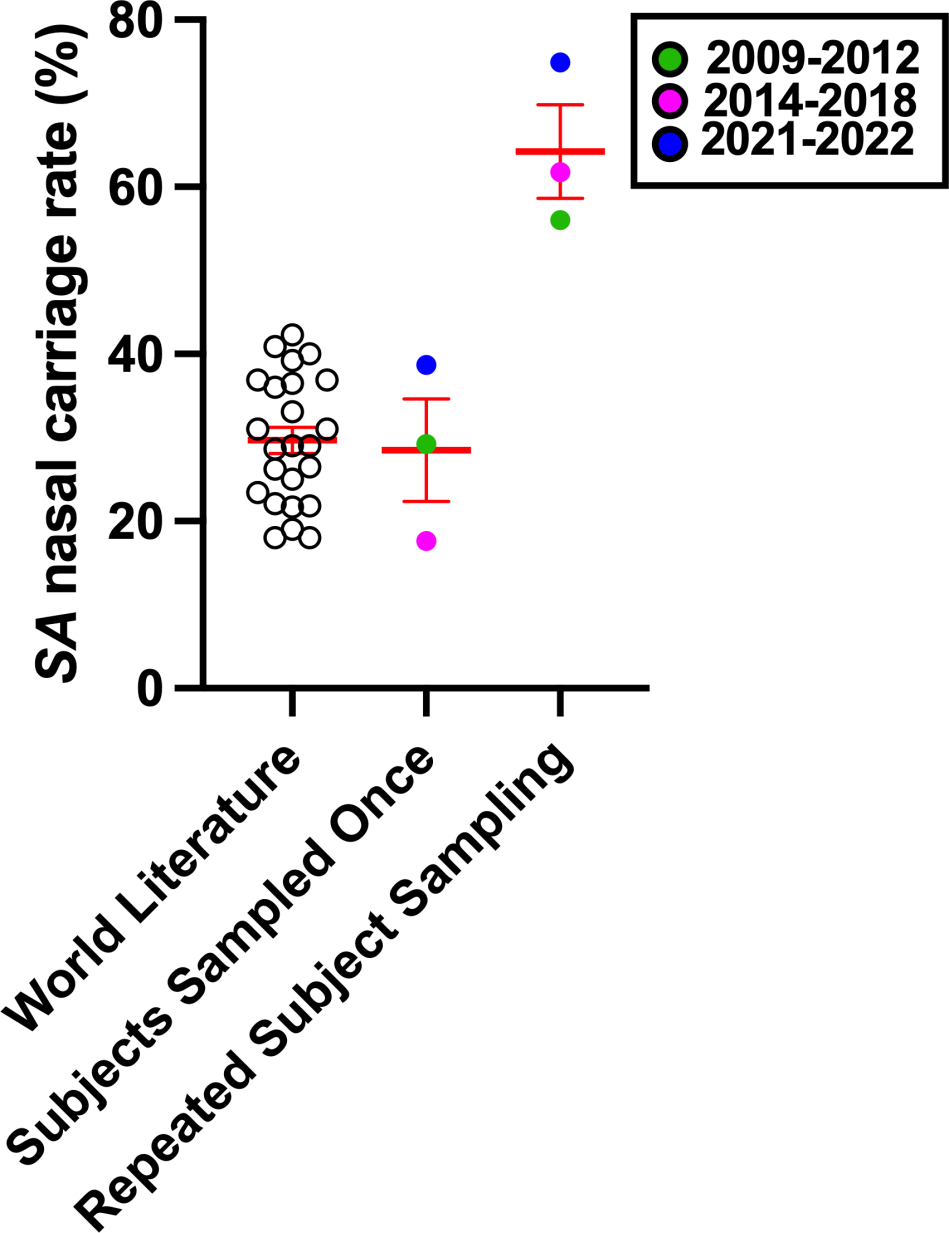
Repeated sampling reveals a SA nasal carriage rate exceeding 60% in healthy adults. Most SA nasal carriage studies report carriage rates based on once or twice sampling of individuals over the duration of weeks. Twenty-four reports from recent world literature are consistent with our group’s observations of a ~30% human SA nasal carriage rate when subjects were swabbed once. Longitudinal sampling over months or years revealed an average carriage rate of 64.2%.

Nasal swabs were collected from university students and staff (13 males, 18 females, average age 26) over several months spanning July 2021 through June 2022. Figure 1 illustrates the participant details, study duration, and experimental overview. As a first-pass assessment of SA carriage rate, status was determined using colony morphology on agar plates, evaluated the next morning (18–24 h) after sample collection. In this (presumed) healthy population, 12 of 31 (38.7%) noses contained culturable SA in at least one nostril swab. Figure 2 shows global SA carriage rates reported for non-clinical/non-hospitalized subjects in relation to the current study, as well as two of our previous reports of subjects evaluated in 2009–2012 (24) and 2014–2018 (38, 47). When subjects were merely screened or sampled at two time points (most reports in the world literature), we and others determined an average nasal SA carriage rate of approximately 30%. We previously discovered, however, that all noses revealed detectable SA DNA when samples were depleted of human DNA prior to nasal microbiome analysis—even noses that were determined by agar plating to never contain SA CFU (38). Therefore, in this study, we incubated a portion of the swab fluids overnight prior to repeat agar plating (termed second overnight culture in Fig. 1) and SA identification and enumeration. Upon evaluation of these cultured swabs, in addition to sample collections repeated weekly over months, we determined that 23 of the 31 subjects (74.9%) exhibited verified nasal SA CFU detected at least once. Taken together, longitudinal sampling of subjects by our laboratory demonstrated that even healthy adults are nasally colonized with SA at an average rate of 64.2% (Fig. 2). Because SA nasal carriage significantly correlates with infection in most hospital and NICU settings worldwide (1, 48, 49), better SA decolonization strategies need development. Topical nasal mupirocin has proven effective at clearing SA in the short term and reducing post-surgical complications (50); however, mupirocin resistance is inevitable (51, 52).

### Identification of species associated with SA culture-negative noses

We previously showed that select *Gammaproteobacteria* are enriched in noses wherein SA was not detected by culture (38). Here, we collected nasal swabs from 31 individuals twice weekly. With the first collection, samples were evaluated by culture on different agars (CHROMagar SA, MacConkey II, TSAII, and Chocolate II) with the intent of identifying SA as well as G-negative species and fastidious species not often identified and described. With the second collection (2–3 days later), swab samples were collected and preserved for microbial DNA profiling. After several weeks of longitudinal sampling, it was apparent that 13 individuals could be categorized as “persistent” carriers because they presented culturable and often high SA load (>10,000 SA CFU/swab) in over 90% of weekly sample collections. The remaining subjects either never demonstrated SA colonies, exhibited lower levels (<100 SA CFU/swab) of SA once or twice, or seemingly switched between detectable and undetected SA (termed “intermittent” carriage). Differential agar plating of samples from these subjects versus persistent carriers revealed colonies on MacConkey II (selective for Gram-negative species) agar 70% of the time, while 10 of 13 SA carrier subjects never presented colonies on MacConkey II. This indicated that Gram-negative species are more likely to be detected in non-SA samples, while SA-positive samples are dominated (in culture) by Gram-positive bacteria. Morphologically unique colonies from intermittent and non-carriers were proliferated overnight in rich broths, biobanked, and identified via 16S rRNA gene sequencing. Corroborating our previous study (38), the following species from the *Gammaproteobacteria* class were collected from intermittent-negative and non-carriers of SA: *Klebsiella* spp. (*aerogenes*, *variicola*, *pneumoniae*), *S. marcescens*, *E. hormaechei*, *E. coli and E. fergusonii*, *R. ornithinolytica*, and *P. rodasii*. For *Firmicutes*, *Streptococcus mitis/oralis* (16S sequencing didn’t discern *S. mitis* from *S. oralis* reproducibly) was collected from nine individuals who never presented SA colonies. Additional species collected from demonstrated non-SA carrier hosts were *C. accolens*, *D. pigrum*, *Bacillus* (*tropicus, paramycoides, koreensis*), and non-aureus *Staphylococcus* (*haemolyticus, lugdunensis, pasteuri*). *S. epidermidis and S. warneri* were collected from both carriers and non-carriers of SA.

Microbial DNA profiling results are shown in Table 1. Nasal swabs (left and right nostrils pooled) from 13 persistent SA carrier subjects were compared to 31 swab samples from intermittent and SA culture-negative hosts (14 subjects, 1–4 sampling dates each). The methodology utilized was an “off the shelf” shotgun metagenomic sequencing service (Illumina platform) that included DNA extraction but not depletion of host (human) DNA. This approach yielded shallow microbial profiling results (Table 1). SA was not detected in any intermittent or non-carrier sample and was detected in only 9 of 13 (69.23%) culture-defined persistent samples. *S. epidermidis*, *Corynebacterium* spp., and *C. acnes* were detected in all noses, as expected. *D. pigrum* was detected only in the intermittent and SA culture-negative group, corroborating our previous study (38) and supporting many others suggesting that *D. pigrum* may prevent SA nasal carriage (34, 53–55). *S. lugdunensis* was detected in 5 of 44 samples (11.4%); *S. mitis/oralis* was detected in 8 of 44 samples (18.2%), and *Gammaproteobacteria* (e.g., *Klebsiella* spp. and *S. marcescens*) weren’t detected well at all despite being cultured from (collectively) over a dozen subjects. This suggests that preferred methods for profiling nasal microbiomes at the species level involve mammalian DNA depletion prior to shotgun short-read sequencing, as we presented previously (38), or the implementation of 16S rRNA gene amplification and long-read approaches that enable species-level resolution (e.g., PacBio [56]). This view, that standard shotgun metagenomics workflows need optimization to improve bacterial detection, is well-discussed by Bjornber et al. (57).

**TABLE 1:** Nasal microbial DNA composition of persistent carriers versus culture-defined intermittent and non-carriers of *S. aureus*^*d*^

	Persistent nasal SA		Intermittent and culture-negative for SA	
Genus and species	Avg. relative abundance (%)	% Colonized noses (*n* = 13)	Avg. relative abundance (%)	% Colonized noses (*n* = 31)
*Staphylococcus* (sum):	37.42	92.31	6.40	80.65
*S. aureus*^*a*^	28.08	69.23	Und	0
*S. capitis*	0.47	7.69	0.231	41.94
*S. epidermidis*	8.72	61.54	5.89	77.72
*S. hominis*	Und	0	0.06	3.23
*S. lugdunensis*	0.14	7.69	0.26	12.90
*Corynebacterium* (sum):	14.96	69.23	51.94	100
*C. accolens*	3.65	30.77	16.76	87.10
*C. segmentosum*	3.09	46.15	4.90	64.52
*C. aurimucosum*	0.46	30.77	0.78	41.94
*C. pseudodiphtheriticum*	Und	0	2.90	19.35
*C. striatum*	Und	0	0.15	12.90
*C. kefirresidentii*	3.45	61.54	7.32	74.19
*C. simulans*	Und	0	0.27	6.45
*C.* unclassified	3.87	38.46	15.3	70.96
*Lawsonella clevelandensis*	3.92	53.85	8.29	93.55
*Cutibacterium acnes*	30.28	92.31	16.08	87.10
*Cutibacterium avidum*	0.20	23.08	0.19	22.58
*Cutibacterium granulosum*	0.50	38.46	1.02	74.19
*Dolosigranulum pigrum*	Und	0	6.86	35.48
*Streptococcus* (sum):	0.21	23.08	0.78	38.71
*S. mitis^b^*	0.09	15.38	0.35	16.13
*S. oralis^c^*	Und	0	0.08	3.23
*S. pneumoniae*	0.05	15.38	0.10	12.90
*S. pseudopneumoniae*	0.03	7.69	0.14	12.90
*S. salivarius*	<0.01	7.69	Und	0
*S. sanguinis*	<0.01	7.69	0.11	12.90
*S.* unclassified	0.03	7.69	<0.01	3.23

^*a*^ *S. aureus* DNA was detected at a lower rate than *S. aureus* CFUs. SA CFUs were used to determine SANC status.

^*b*^ *S. mitis* was cultured from 4 of 10 non-carrier nasal swabs tested.

^*c*^ *S. oralis* was cultured from 3 of 10 non-carrier nasal swabs tested.

^*d*^ Und = indicated bacterial DNA not detected in any nose, as determined by shotgun metagenomic sequencing.

### Survival of SA in nasal epithelia is potently inhibited by *Klebsiella* spp.*, S. marcescens*, and *E. hormaechei*

**Fig 3:**
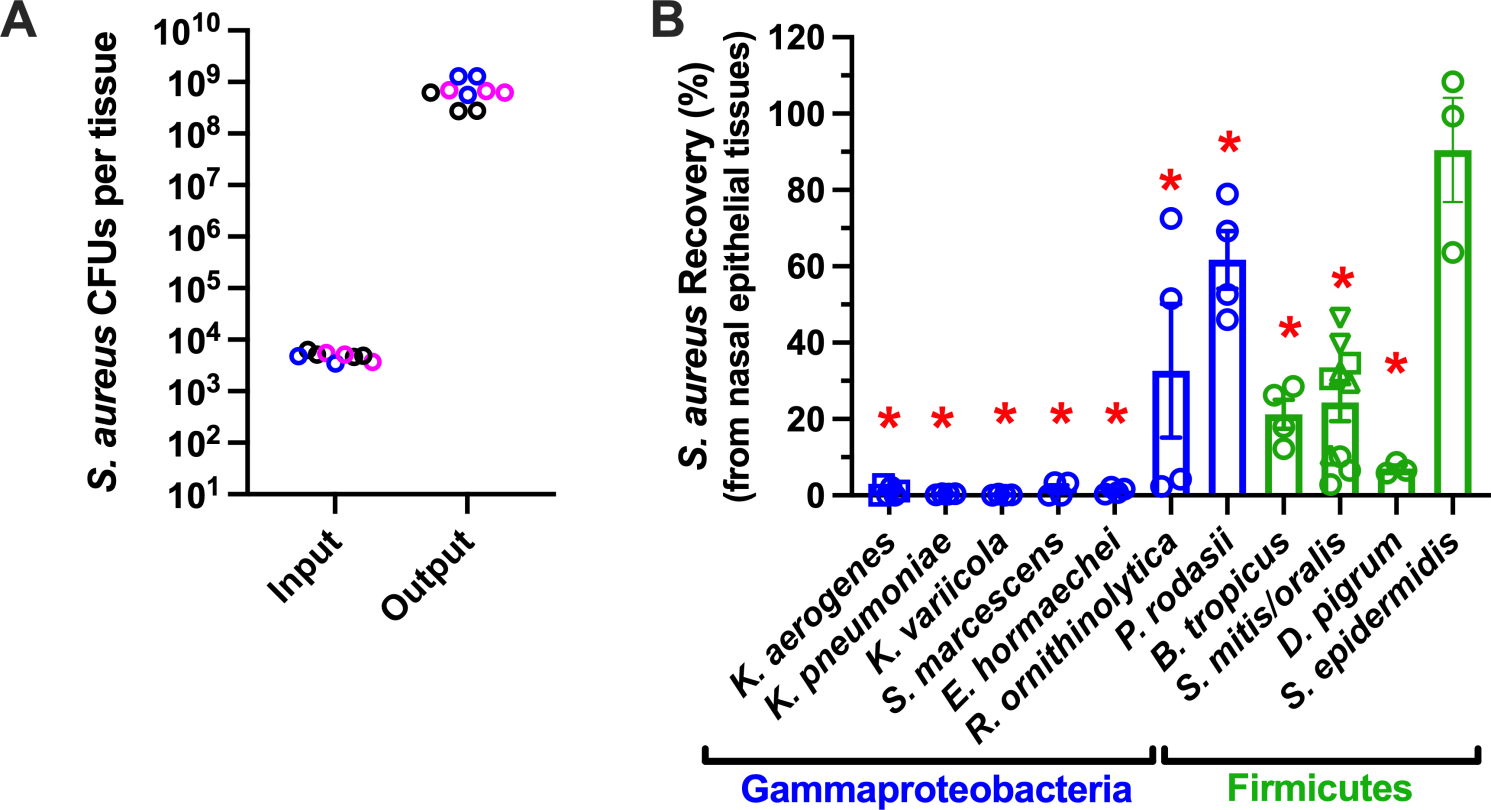
Potent inhibition of SA by diverse *Gammaproteobacteria*. SA competition assays were performed on polarized primary human nasal epithelia that were pre-treated one day prior with nasal commensal species *C. accolens* and *C. acnes*. (**A**) Growth of three unique nasal strains of SA (USA300, D547, D512; three experiments each, strains distinguished by color), on pre-colonized nasal epithelia, presented as input versus output CFU/well at 24 h. (**B**) SA recovery (%) for tissue treatments containing 50% SA + 50% indicated strain versus growth of SA alone on pre-colonized epithelia. *P < 0.0001 versus SA incubated alone on pre-colonized epithelia (100% recovery). Bars and error bars represent mean ± SEM; *n* = 3–10 for each species tested in competition with SA. Shapes indicate independent experiments, with circles, squares, and triangles representing distinct nasal isolates of *K. aerogenes* and *S. mitis/oralis*.

Nasal bacteria (11 species/25 isolates) were tested for activity against SA using a physiologically relevant environment in which SA thrives. Bacterial mixtures (5,000 SA CFU + 5,000 CFU competitor) were placed on primary human nasal epithelia that were polarized on porous Transwell inserts and pre-treated with nasal commensal species *C. accolens* and *C. acnes* one day prior. Without antibiotics in the nasal epithelia or underlying medium, which contained growth factors, the SA inoculum proliferated over 10,000-fold in 24 h (Fig. 3A). *Klebsiella* spp. and *E. hormaechei* inhibited SA recovery to <1%; *S. marcescens* and *D. pigrum* inhibited SA recovery to <10%; and *B. tropicus* and *S. mitis/oralis* strains limited SA recovery to <50% compared to application of SA alone (Fig. 3B). Multiple nasal strains of *S. epidermidis* exhibited no inhibitory effect on SA. It should be noted that *R. ornithinolytica* and *P. rodasii* were difficult to recover and enumerate, and it is possible that their diminished potency compared to other *Gammaproteobacteria* was due to less metabolic activity on the nasal tissues. Likewise, *D. pigrum* was not able to be recovered following application to nasal tissues, although its apparent potency against SA warrants further investigation once nutrient and/or cohabitating bacterial requirements are established. *B. tropicus* efficacy was inconsistent, as different results were observed with different donor isolates and input ratios with SA (not shown), and it was not investigated further. *S. mitis* and *S. oralis* potencies were isolate dependent, and these species are being investigated in a separate project. *E. coli and E. fergusonii* did not propagate well in SNM-HS and were not evaluated on tissues after failing to compete with SA in tissue-free screening assays. Taken together, Fig. 3 demonstrates potent anti-SA activity by *Klebsiella* spp. (*aerogenes*, *variicola*, *pneumoniae*), *S. marcescens*, and *E. hormaechei* in an environment modeling the nasal epithelia.

### Inhibition of nasal SA by *Klebsiella*- and *S. marcescens*-derived factors

**Fig 4:**
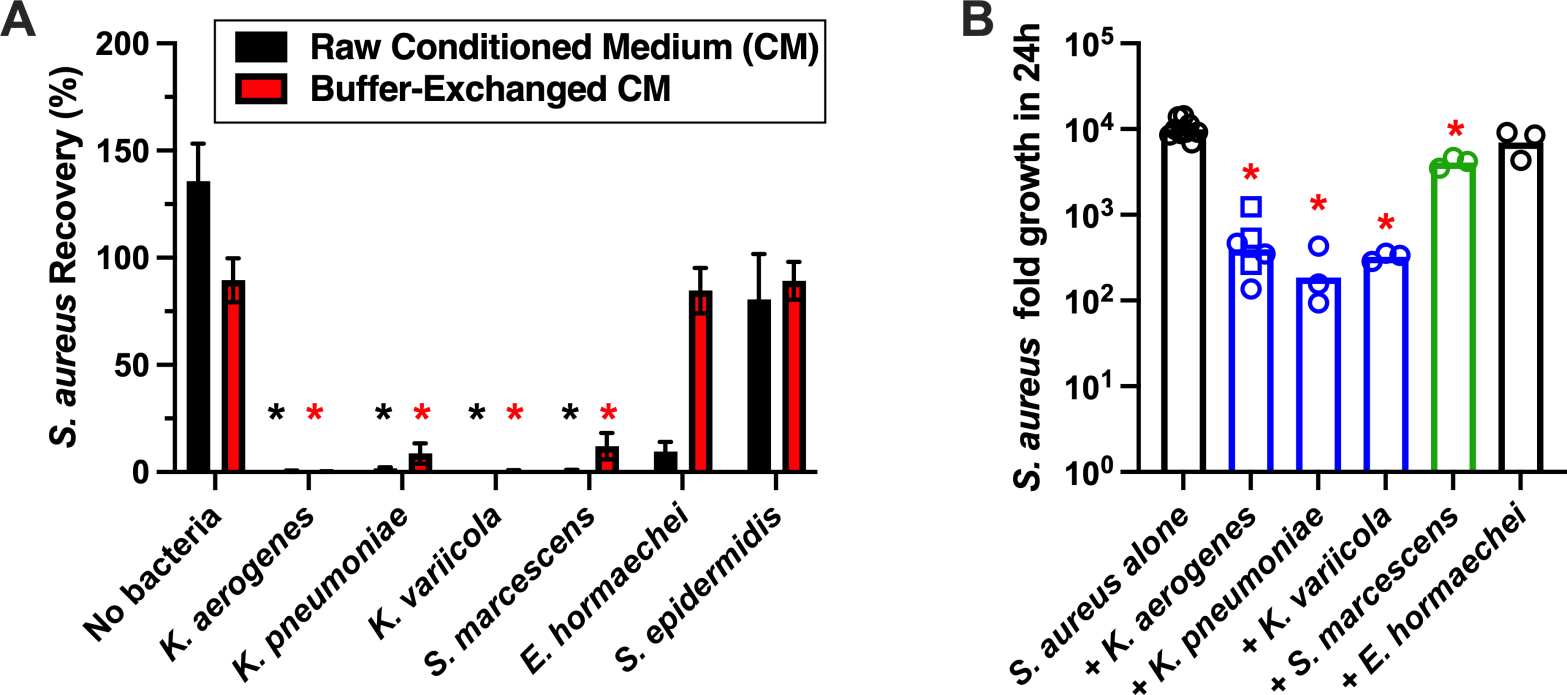
Inhibited growth of SA by *Klebsiella*- and *S. marcescens*-derived factors. (**A**) SA was incubated in raw conditioned medium (CM, black bars) collected from the indicated bacteria cultured for 24 h in simulated nasal medium (SNM-HS), or in bacterial CM that was buffer exchanged with fresh SNM-HS (red bars). SA recovery (%) was measured after 24 h compared to growth in plain fresh SNM-HS. Bars = mean ± SEM; *n* = 4 independent experiments (two each with SA strains D547 and USA300). * indicates *P* < 0.05 compared to SA recovery from SNM-HS. (**B**) SA (strain USA300) fold growth in 24 h when grown alone in rich medium (TSB) compared to when SA was incubated with an equivalent input of the indicated species. Bars = mean ± SEM. Open circles designate independent experiments (*n* = 3 for each competitor bacteria and *n* = 9 for SA). Squares indicate a second nasal isolate of *K. aerogenes*. * indicates *P* < 0.001 versus SA alone.

We next evaluated whether promising competitor species’ activity against SA was contact-independent and reliant on bacterial-derived factors rather than sequestration of nutrients. CM was collected from each bacterium of interest following culture in SNM-HS for 24 h, and a portion of each CM was buffer-exchanged with fresh SNM (e.g., proteins/peptides from CM preserved but nutrient/metabolite background equivalent to fresh SNM-HS. Prior to testing SA growth, CMs were filter-clarified and confirmed free of bacterial CFUs by agar plating. The growth of two different strains of SA (D547 [ST5] and USA300 [ST8]) was evaluated in fresh SNM-HS or prepared CMs after incubation for 24 h. CM from *K. aerogenes*, *K. pneumoniae*, *K. variicola*, and *S. marcescens* inhibited SA detection potently, regardless of buffer exchange, while CM from *E. hormaechei* and *S. epidermidis* did not affect SA recovery (Fig. 4A). This suggested that secreted proteins from select *Gammaproteobacteria* (e.g., *Klebsiella* spp. and *S. marcescens*) limit SA survival, while *E. hormaechei* may have inhibited SA by sequestering nutrients. To confirm that nutrient limitation was not biasing the SNM-HS-based assays, live bacteria competition assays were performed in rich bacterial medium (TSB), and SA fold-growth was measured. Potent inhibition of SA was again observed for *K. aerogenes*, *K. pneumoniae*, and *K. variicola*, along with a more modest reduction observed for *S. marcescens,* while *E. hormaechei* showed no effect (Fig. 4B). Collectively, these data are consistent with the concept that nasal carriers of *Klebsiella* spp. and *S. marcescens* are less likely to have SA detected in routine screens. The observation that nasal *Gammaproteobacteria* abundance is inversely proportional to SA abundance is corroborated by several recent observational clinical studies of infants and children, as well as adult diabetic foot osteomyelitis (58–61).

### Role of iron limitation in *Klebsiella* spp.-mediated activity against SA

**Fig 5:**
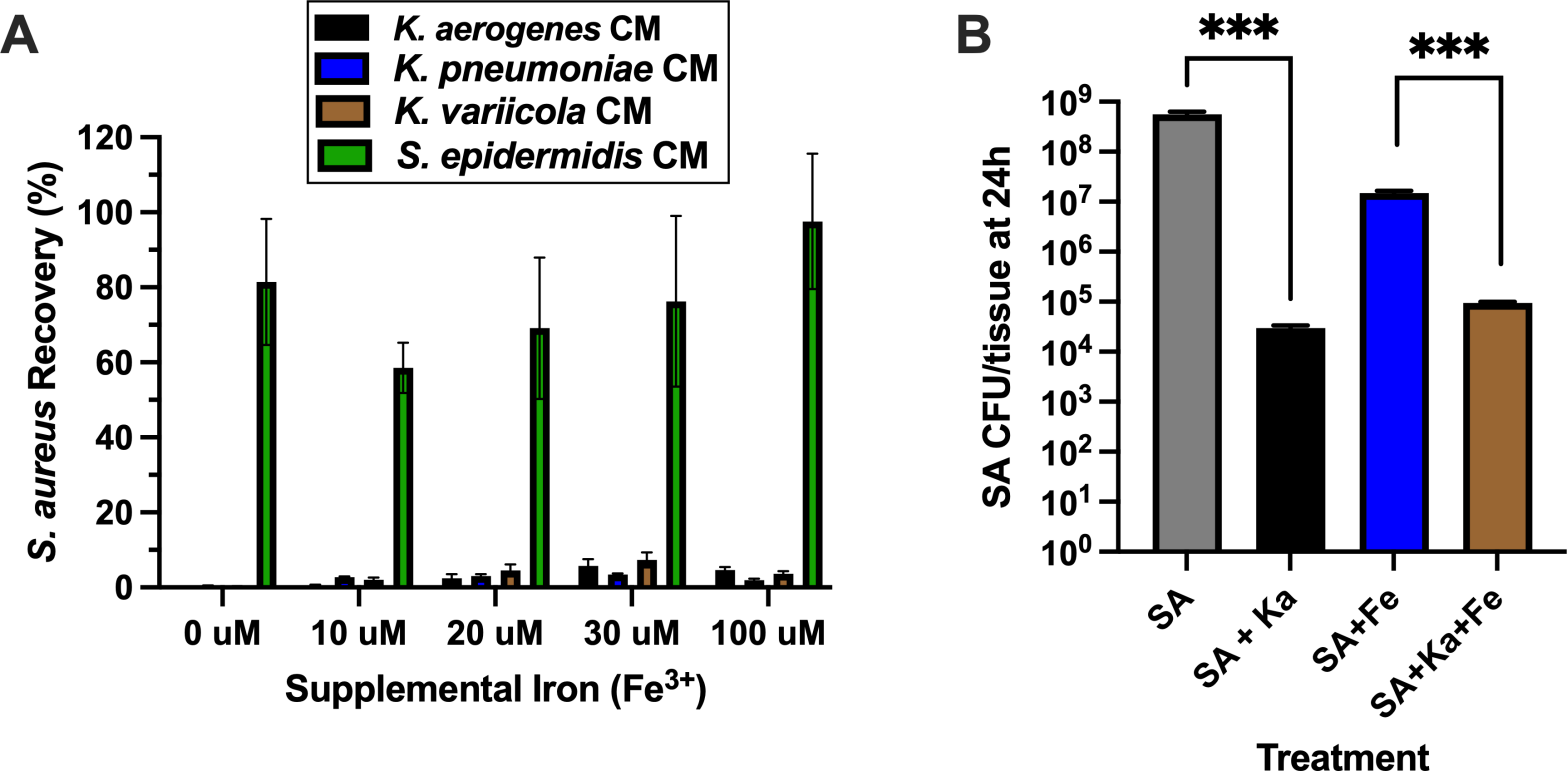
*Klebsiella* spp.-mediated inhibition of nasal SA is independent of iron availability. (**A**) The indicated CMs were supplemented, or not, with 10–100 uM iron, and growth of SA (strain D547) was monitored in comparison to growth in fresh SNM-HS. Bars = mean ± SEM SA recovery (%); *n* = 3 independent experiments. (**B**) Polarized nasal epithelia containing a mixture of commensals (*C. accolens*, *C. acnes*, and *S. epidermidis*) were treated with 5,000 CFU of SA alone or SA+*K. aerogenes* (Ka), with or without supplemental iron (Fe) as indicated. Bars = mean ± SEM; *n* = 3 independent experiments. *** indicates *P* < 0.001.

Since SA is known to be susceptible to iron limitation (62, 63), we next tested whether supplementing CM with iron would reverse the observed inhibition of SA recovery. Adding 10–100 µM Fe^3+^ (20–30 µM typical in nasal fluids [64]) to *Klebsiella* spp. CMs did not restore SA detection, and overall recovery of SA was still <10% compared to SA recovery from control SNM-HS or CM collected from *S. epidermidis* (Fig. 5A). When nasal tissues were supplemented with 30 µM Fe^3+^ during treatment with SA or the mixture of SA+*K. aerogenes*, the overall trend that *K. aerogenes* reduced SA recovery by over 100-fold was not reversed (Fig. 5B). It is not known precisely why iron supplementation reduced SA recovery from tissues that weren’t administered *K. aerogenes* (blue versus gray bar in Fig. 5B), but more commensal species CFUs were observed when we enumerated SA collected from the “SA+Fe” tissues (blue bar, Fig. 5B). This suggested that application of Fe^3+^ benefitted one or more of the commensal species (*C. acnes*, *C. accolens*, and *S. epidermidis*) at the expense of SA growth during the 24 h treatment period. Further studies and better multi-species discernment and enumeration methods need to be developed in order to explain this difference in SA detection.

When we subjected *K. aerogenes* CM to LC-MS/MS analysis, the peptides identified included the list shown in Table 2. We have not yet been successful at isolating these peptides from *K. aerogenes* CM and analyzing them individually; however, these peptides warrant further investigation in the context of inhibiting SA survival in the nasal mucosa. It should be noted that tonB-dependent receptor fragments (2 and 6 kD spots) were also identified from unique gel spots observed in *K. aerogenes* CM (not shown). TonB-dependent outer membrane receptors capture ferric siderophores to begin iron uptake. Specific studies on *K. aerogenes* siderophores are not abundant, while over 98% of *K. pneumoniae* and *K. oxytoca* strains have been demonstrated to produce and secrete enterobactin, which has a high affinity for Fe^3+^ (65, 66). The influence of *Klebsiella* siderophores on the nasal mucosa and SA survival needs further investigation. A recent study of the medicinal plant *Kalanchoe blossfeldiana* also showed that extracts from plant-isolated *K. aerogenes* demonstrated anti-SA activity (67), which is in line with the results presented here.

**TABLE 2:** *K. aerogenes*-derived peptides identified by LC-MS/MS analysis of bioactive (anti-*S. aureus*) conditioned medium^*a*^

Protein name	Protein accession no.^*b*^	Peptide sequence
Lysis protein OS = Klebsiella aerogenes OX = 548 GN = BXQ27_34070 PE = 4 SV = 1	A0A1V3YHN2_KLEAE	MIFSGFLLVACQANYIR
Putative porin OS = Klebsiella aerogenes OX = 548 GN = SAMEA2053898_01674 PE = 4 SV = 1	A0A7Z8TN76_KLEAE	MLRFYVTGGQVNNEHTAK
Entericidin B membrane lipoprotein OS = Klebsiella aerogenes OX = 548 GN = ecnB PE = 3 SV = 1	A0A094WZ39_KLEAE, A0A0H3FVB4_KLEAK	GIGEDISDGGSAISGAATK
Phage lysis protein OS = Klebsiella aerogenes OX = 548 GN = BXQ27_12255 PE = 4 SV = 1	A0A1V3Z7Q1_KLEAE	MSLCPMPGSDPKTNGDLSADIR
Type VI secretion system protein ImpH OS = Klebsiella aerogenes OX = 548 GN = impH PE = 4 SV = 1	A0A3S4NAT6_KLEAE	MGREAQPPHSR
Iron-sulfur cluster assembly protein CyaY OS = Klebsiella aerogenes OX = 548 GN = cyaY PE = 3 SV = 1	A0A447WIB5_KLEAE	QGGYHFDLK
Ferric uptake regulation protein OS = Klebsiella aerogenes OX = 548 GN = fur PE = 3 SV = 1	A0A3S4KZJ5_KLEAE	MTDNNTALKK
LamB type porin OS = Klebsiella aerogenes OX = 548 GN = scrY_6 PE = 3 SV = 1	A0A447WEQ1_KLEAE	MVSGMRAK
Klebicin B OS = Klebsiella aerogenes OX = 548 GN = ASV18_24475 PE = 3 SV = 1	A0A0W2DKY3_KLEAE	KYQDLQQSIK
TonB-dependent siderophore receptor OS = Klebsiella aerogenes OX = 548 GN = HV331_04175 PE = 3 SV = 1	A0A7D7JJQ3_KLEAE	GANSLLNGAASSGVGGMINLEPKR

^*a*^ OS, organism species; OX, organism taxonomy (NCBI ID for *K. aerogenes *= 548); GN, gene name or database matched accession number; PE, Mascot protein expectation score; SV, ratio of the number of amino acids in identified peptide to the number of amino acids in the total protein sequence (SV = 1 indicates 100% sequence coverage).

^*b*^ Protein accession numbers can be found at https://www.uniprot.org/uniprotkb.

### Conditioned medium from *K. aerogenes* cultures exhibits anti-SA activity mediated by cationic proteins

**Fig 6:**
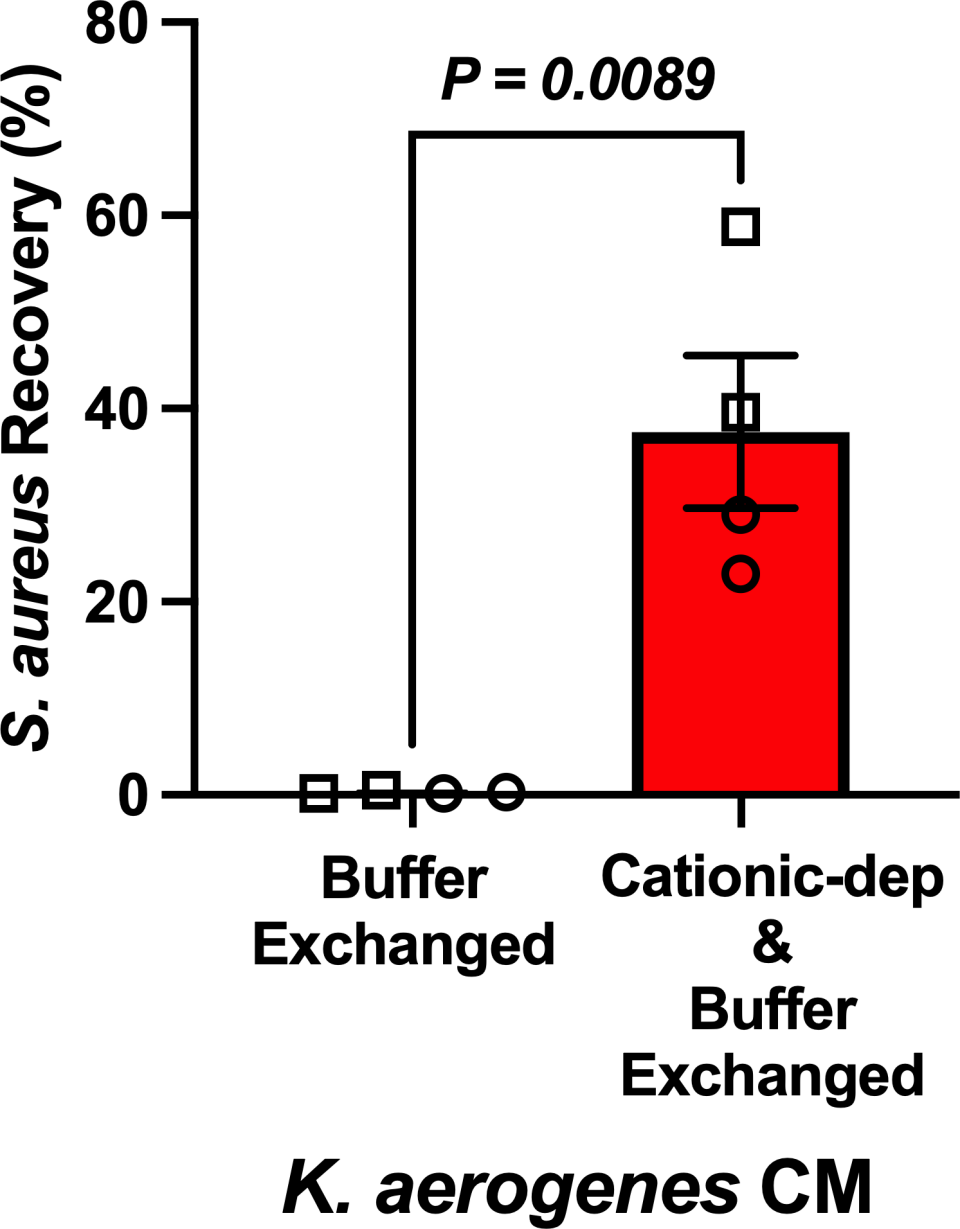
SA inhibition by *K. aerogenes* is at least partially dependent on cationic proteins or peptides. SA (strain D547) growth was assessed in buffer-exchanged *K. aerogenes* CM or cationic protein-depleted, buffer-exchanged *K. aerogenes* CM compared to growth in plain SNM-HS (control), and % recovery was calculated. Bars = mean ± SEM; *n* = 4 independent experiments. Open circles and squares indicate unique *K. aerogenes* nasal isolates.

To further evaluate potential bioactive peptides and/or proteins in *K. aerogenes* CM, we next measured SA recovery from CM that was buffer-exchanged with fresh SNM or CM that was cationic protein-depleted and then buffer-exchanged, compared to plain SNM-HS that underwent the same manipulations in parallel. Significantly greater SA recovery was observed in the cationic-depleted fluid compared to buffer-exchanged *K. aerogenes* CM (Fig. 6). SA recovery from control (unconditioned medium) manipulated in parallel to *K. aerogenes* CM was not statistically different from SA recovery from plain SNM-HS, suggesting that *K. aerogenes*-derived cationic factors impacted SA recovery. Ongoing efforts in our laboratory to isolate individual *K. aerogenes* peptides with anti-SA activity have not yet been successful, likely due to the nature of antimicrobial peptides, which are typically small, redundant, and adherent (to other proteins as well as lab plastics and centrifugal filters). Many antimicrobial peptides also function synergistically, meaning that multiple peptides may not achieve full antimicrobial function individually but are effective in concert (68–70). We determined previously that the anti-SA activity of *K. aerogenes* CM was heat-stable but was lost following proteinase K treatment (38). We also demonstrated that bioactive CM fractions from *K. aerogenes* were composed of only 39.8 ± 11.1 µg/mL of total protein, suggesting that anti-SA protein(s) or peptide(s) may function in the nM or low μM range (38). Collectively, the presented data suggest that novel and natural inhibitors of SA nasal carriage may exist in carriers of select *Gammaproteobacteria,* including *Klebsiella* spp.

### Summary, study limitations, and conclusion

In summary, this study highlights a connection between nasal carriers of diverse *Gammaproteobacteria*, particularly *Klebsiella* spp., and conferred resistance to nasal SA. While a limitation of our study is the small sample population from a single site, our findings are in line with seminal reports relating nasal microbiota and SA nasal carriage, and many larger studies that focused more on bacterial DNA levels than bacterial functional interactions (34, 71–73). Notably, our findings reinforce the concept that typical nasal screening (one-time swab) underestimates the SA nasal carriage rate. Indeed, we found that even in a healthy collegiate population, the true carriage rate observed using repeated weekly sampling was 74.9% for the current cohort and 6,064.2% across three different studies performed between 2009 and 2022 (Fig. 2). We also established physiologically relevant assays for evaluating SA competitors in the nasal milieu and demonstrated that *K. aerogenes*, *K. variicola*, *K. pneumoniae*, and *S. marcescens* inhibited SA detection without direct contact, irrespective of physiological iron and total nutrient availability (Fig. 3 to 5). *K. aerogenes*-derived cationic peptides or small proteins were suggested to be at least partially responsible for anti-SA activity (Fig. 6). Taken together, this study underscores the relevance of Gram-negative species in noses that naturally resist SA colonization and brings attention to the need for better detection methods for low-abundance bacteria. The nasal microbiome is highly variable yet exhibits relatively low species diversity that is likely attributed to low nutrient supply (36). *Staphylococcus* and *Streptococcus* factors influencing SA carriage have received more attention than *Proteobacteria*, likely due to the ease of isolation and propagation using standard methods (36). Similarly, standard microbial DNA profiling services have not been optimized for nasal samples that are rich in human DNA, and *Proteobacteria* such as *Klebsiella* and *Moraxella* spp. (predominant in CST-6 and associated with SA non-carriage [34, 38]) are not often detected relative to *Actinobacteria* and *Firmicutes* (Table 1). However, we and others demonstrate that nasal carriers of *Klebsiella* spp., classified previously as nasal CST-2 (34), represent a group warranting more investigation in terms of which genetic, dietary, immune, and environmental factors associate with this nasal microbiome type. To date, the advertisement and widespread use of probiotics is owed to the idea that raising the number of “beneficial” microbes colonizing the gastrointestinal tract offers considerable benefit to the host (74). While the mechanisms underlying the protective nature of *Klebsiella* spp. versus SA at mucosal surfaces such as the nose remain elusive, the concept of microbiome editing to prevent infection gained traction with respect to SA carriage when ingestion of *Bacillus subtilis* significantly reduced SA in both stool samples and nasal swabs (75). While the link between intestinal and nasal carriage of SA is not yet fully understood, a better understanding of nasal microbiota dysbiosis and mechanisms of microbiome-mediated exclusion of nasal SA will enable better infectious disease care in vulnerable individuals.
